# The relationship between immediate postmastectomy reconstruction modalities and survival benefits in patients with triple negative breast cancer

**DOI:** 10.1002/cam4.5166

**Published:** 2022-09-15

**Authors:** Luyao Dai, Hanxiao Cui, Yuanhang Bao, Liqun Hu, Zhangjian Zhou, Meng Wang, Shuai Lin, Hao Wu, Huafeng Kang, Xiaobin Ma

**Affiliations:** ^1^ Department of Oncology The Second Affiliated Hospital of Xi'an Jiaotong University Xi'an Shaanxi China; ^2^ School of Basic Medical Sciences, Xi'an Key Laboratory of Immune Related Diseases Xi'an Jiaotong University Xi'an Shaanxi China

**Keywords:** breast cancer‐specific survival, overall survival, reconstruction, surveillance, epidemiology, and end results, triple negative breast cancer

## Abstract

**Introduction:**

Immediate postmastectomy reconstruction for breast cancer has been widely used due to its unique esthetic and psychological effects. However, no other population‐based study has investigated the effects of different reconstruction types on the survival in patients with triple negative breast cancer (TNBC).

**Methods:**

We selected patients who met the eligibility criteria from the Surveillance, Epidemiology, and End Results cancer registry (*N* = 9760). We then assessed the effect of different reconstructive surgical approaches (implant, autologous, implant and autologous combined reconstruction) on the overall survival (OS) and breast cancer‐specific survival (BCSS) by using the Kaplan–Meier survival curve and Cox proportional hazard regression analyses. The nomograms were used to predict OS and BCSS. And the competitive risk model was used to assess breast cancer‐specific death (BCSD) and non‐breast cancer‐specific death (NBCSD).

**Results:**

Statistical analysis suggested that the three reconstruction methods had better OS and BCSS with lower hazard than mastectomy alone (log‐rank test, *p* < 0.05). Multivariate stratified analysis showed that patients aged 40–60 years had the greatest improvement in OS (Adjusted hazard ratio [AHR], 0.646; 95% Confidence Interval [CI], 0.439–0.950; *p* = 0.026) with combined reconstruction. BCSS could be improved only by implant reconstruction (AHR, 0.672; 95% CI, 0.514–0.878; *p* = 0.004). In addition, autologous reconstruction (AHR, 0.570; 95% CI, 0.350–0.929; *p* = 0.024) and implant reconstruction (AHR, 0.538; 95% CI, 0.339–0.853; *p* = 0.008) improved OS in patients >60 years of age. The survival prediction model quantified the survival benefits of TNBC patients undergoing different surgeries. Moreover, the C‐indexes showed the good predictive ability of the nomograms.

**Conclusions:**

Our results suggest that for TNBC patients, there is a survival benefit of immediate postmastectomy reconstruction compared with mastectomy alone. Among them, implant reconstruction has the most obvious advantage.

## Graphical abstract

Kaplan–Meier survival curves and log‐rank tests were used to evaluate the effect of different reconstruction methods (implant, autologous, implant and autologous combined reconstruction) on the overall survival (OS) of triple negative breast cancer patients. All three reconstruction methods had higher OS and lower risk than mastectomy alone (*p* < 0.0001). Among the three reconstruction methods, autologous reconstruction has the lowest survival rate and the highest risk.

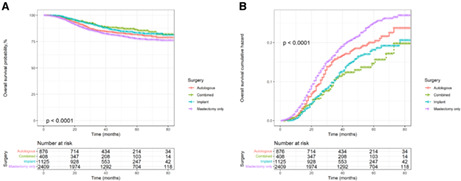

## 1 INTRODUCTION

Breast cancer (BC) is the most common cancer among women worldwide. It is also the leading cause of cancer‐related death in women, making it one of the highest burden of cancers in the world.^1^, ^2^, ^3^ According to the presence of estrogen receptor (ER), progesterone receptor (PR), and human epidermal growth factor receptor 2 (HER2), as well as Ki67 status, BC can be classified into different molecular subtypes.^4^ Among them, triple negative breast cancer (TNBC) does not have ER, PR, and HER2 expression, accounting for approximately 15% of BCs.^5^ Research showed that for tumors of all sizes, TNBC had a higher hazard ratio (HR) and a higher risk of recurrence and metastasis than all other subtypes.^6^, ^7^ This uniqueness often indicates that patients will have a poor prognosis and bring unique treatment challenges.^8^

Surgery is the main treatment of early‐stage BC. The traditional surgical method is mastectomy.^9^, ^10^, ^11^ However, the results of mastectomy often greatly affect body image, cause anxiety and depression, and significantly reduce the quality of life.^12^ This is why breast reconstruction is applied. Studies have shown that breast reconstruction can help patients maintain a good physical appearance, achieve good psychosocial outcomes, and benefit from a higher quality of life.^13^, ^14^, ^15^, ^16^, ^17^ There are two main methods of reconstruction: immediate breast reconstruction (IBR) and delayed breast reconstruction (DBR).^18^ Some reports suggested that IBR has become more common than DBR^19^, ^20^ because of better overall cosmetic outcome, higher level of mental health, and lower cost.^21^, ^22^, ^23^, ^24^, ^25^, ^26^, ^27^ Previous population‐based studies have demonstrated an association between breast reconstruction and improved survival.^28^, ^29^ Several studies have shown that breast reconstruction will not damage the detection of local recurrence.^30^, ^31^ Shailesh Agarwal et al.^32^ pointed out that the prognosis of patients undergoing IBR would not be worse than that of patients undergoing mastectomy even though the tumor safety of breast reconstruction is still a controversial topic. Moreover, for patients with more malignant TNBC, whether the survival of patients will be affected by reconstruction is not clear. The purpose of this study was to evaluate the relationship between breast reconstruction methods and overall survival (OS) and breast cancer‐specific survival (BCSS) in patients with TNBC.

## 2 METHODS

### 2.1 Study population selection and data extraction

Data were obtained from the Surveillance, Epidemiology, and End Results (SEER) program records of the National Cancer Institute, using SEER*Stat version 8.3.9.1 (http://seer.cancer.gov/seerstat). We selected female patients diagnosed as TNBC with original primary malignancy between 1975 and 2016 from the SEER database. The participants included patients who underwent simple mastectomy (40–49, 75), modified radical mastectomy (50–59, 63), radical mastectomy (60–62, 64–69, 73–74), and mastectomy (80). The exclusion criteria were as follows: (1) Patients who underwent subcutaneous mastectomy, partial mastectomy or extended radical mastectomy; (2) diagnosis via an autopsy or a death certificate only; (3) with history of other malignancies; (4) survival time was equal to zero; and (5) with missing, borderline, or unknown data. The information in SEER database is publicly available, so the patients' informed consent is not required. The ethics committee of the Second Affiliated Hospital of Xi'an Jiaotong University approved the study.

### 2.2 Statistical analysis

Descriptive statistics were performed for women who underwent mastectomy alone and IBR after mastectomy. The latter type was divided into three subgroups: implant reconstruction, autologous reconstruction (rectus abdominis flap, latissimus dorsi flap, or flap not otherwise specified), and implant reconstruction combined with autologous reconstruction. To simulate randomized controlled trials and balance patient characteristics between groups, we used the nearest neighbor method for 1:1 propensity score‐matching (PSM) method.

The Chi‐squared test was used to examine the differences in patients' clinical characteristics between the mastectomy and reconstruction subgroups. The Kaplan–Meier plots and log‐rank tests were performed to compare the OS and BCSS rates between these surgery groups. For the univariate and multivariate analyses, Cox proportional models were used to estimate the HRs and 95% CI of the survival differences among reconstruction subgroups in special demographic or pathological subgroups. Survival times were measured from the time of diagnosis to breast cancer‐specific death (BCSD), non‐breast cancer‐specific death (NBCSD), date of last follow‐up, or December 2016. A competitive risk model was used to evaluate BCSD and NBCSD in patients undergoing different surgical procedures, as measured by cumulative incidence. In order to individually predict the 1‐year, 3‐year, and 5‐year survival probabilities of patients, the survival prediction model nomograms were plotted at last.

All statistical analyses were performed using the R software (Version 4.0.3; http://www.r‐project.org). The main packages used were “survival”, “survminer” and “rms”. A two‐sided *p* value <0.05 was considered statistically significant.

## 3 RESULTS

### 3.1 Patients' baseline characteristics

**FIGURE 1:**
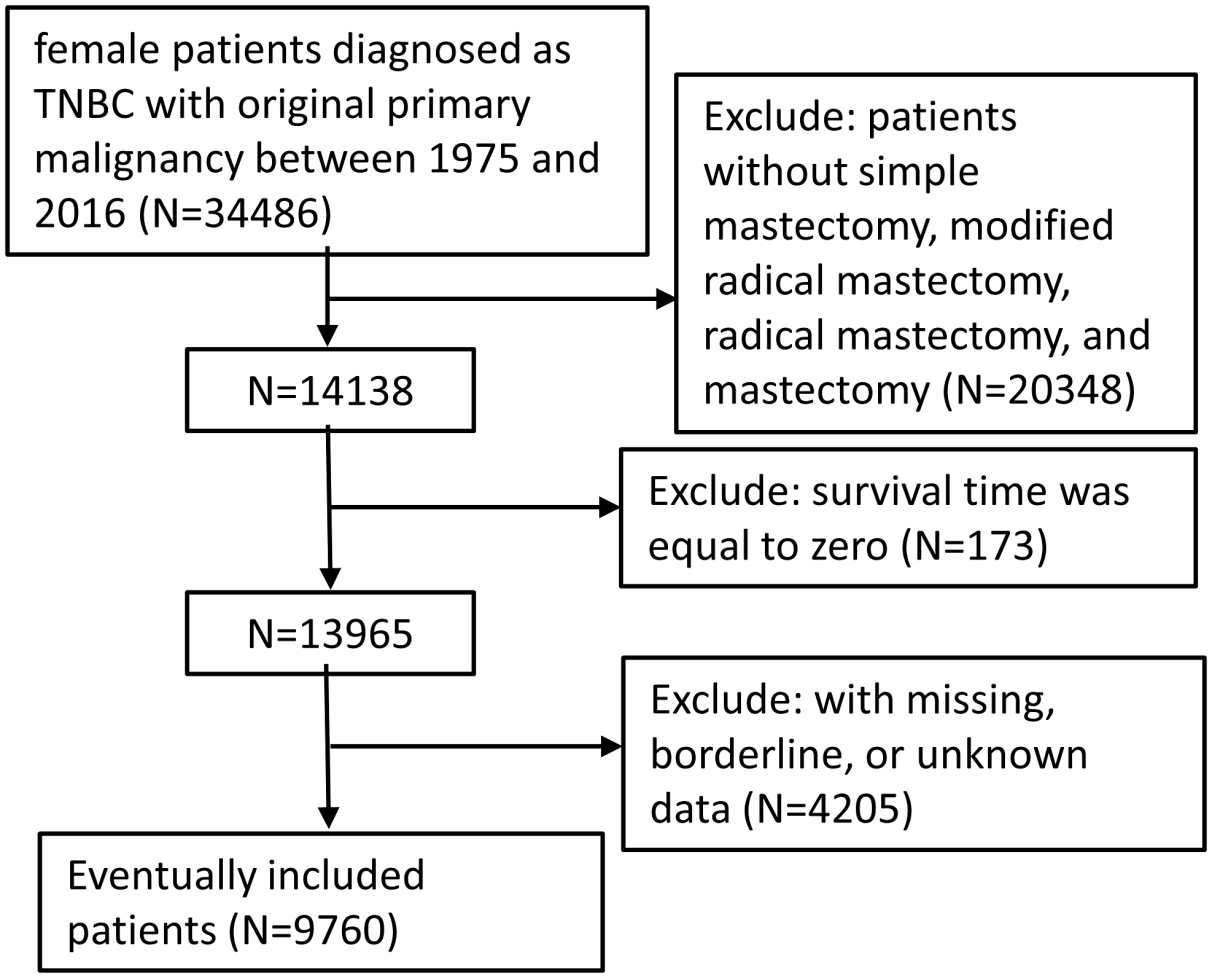
Flow chart of patient inclusion.

Using the inclusion criteria, the present study included 9760 female patients diagnosed with TNBC between 1975 and 2016, who underwent BC surgery (Figure 1). Median follow‐up time for OS and BCSS was 47 months (95% CI, 46–47 months) and 45 months (95% CI, 44–46 months), respectively. Significant distribution differences in age, race, marital status, histologic type, stage, grade, regional nodes positive number, radiation, and chemotherapy were observed between the mastectomy and all reconstruction group (Table 1), but there was no significant difference in grade between mastectomy and the three reconstruction subgroups. Among the included patients, 7351 had mastectomy only, while fewer had undergone breast reconstruction, at 2409. As shown in Table 1, in every age group, there was a higher rate of mastectomy than reconstruction. The majority of patients opting for reconstruction were in the 40–60 age group, and the lowest percentage of patients who opted for mastectomy were <40 years old. In addition to age, patients who chose breast reconstruction were more likely to be white, married, ductal histology type, stage II, grade III, without regional lymph node positivity, no radiation, but with chemotherapy. The characteristics of these patients were consistent with those opting for mastectomy except for age. Of the three reconstruction subgroups, the implant subgroup (1125) had the largest number, followed by the autologous subgroup (876). The combined subgroups (408) had the smallest number. Obviously, there were remarkable similarities in the distribution characteristics of patients among the reconstruction subgroups, which were in accordance with the overall reconstruction population. After excluding the deviation through PSM (Table 2), except that there was still a significant distribution difference in the race of each reconstruction subgroup (*p* = 0.041), the characteristics of the other groups were evenly distributed (*p* > 0.05).

**TABLE 1:** Baseline demographic characteristics of patients with triple‐negative breast cancer

Variable	Mastectomy only	Reconstruction type				P1^a^	P2^b^
		All	Implant	Autologous	Combined		
	No. (%)	No. (%)	No. (%)	No. (%)	No. (%)		
	7351	2409	1125	876	408		
Age, year						<0.001	<0.001
<40	692 (9.4)	530 (22.0)	262 (23.3)	179 (20.4)	89 (21.8)		
40–60	3357 (45.7)	1506 (62.5)	689 (61.2)	561 (64.0)	256 (62.7)		
>60	3302 (44.9)	373 (15.5)	174 (15.5)	136 (15.5)	63 (15.4)		
Race recode						<0.001	<0.001
White	5177 (70.4)	1878 (78.0)	887 (78.8)	659 (75.2)	332 (81.4)		
Black	1454 (19.8)	390 (16.2)	163 (14.5)	169 (19.3)	58 (14.2)		
Other	720 (9.8)	141 (5.9)	75 (6.7)	48 (5.5)	18 (4.4)		
Marital status						<0.001	<0.001
Married	3947 (53.7)	1595 (66.2)	743 (66.0)	567 (74.7)	285 (69.9)		
Unmarried^c^	3404 (46.3)	814 (33.8)	382 (34.0)	309 (35.3)	123 (30.1)		
Histologic type						<0.001	0.004
Ductal	6203 (84.4)	2106 (87.4)	980 (87.1)	766 (87.4)	360 (88.2)		
Other	1148 (15.6)	303 (12.6)	145 (12.9)	110 (12.6)	48 (11.8)		
Stage						<0.001	<0.001
I	1567 (21.3)	791 (32.8)	376 (33.4)	282 (32.2)	133 (32.6)		
II	3423 (46.6)	1237 (51.3)	593 (52.7)	433 (49.4)	211 (51.7)		
III	2045 (27.8)	351 (15.6)	144 (12.8)	147 (16.8)	60 (14.7)		
IV	316 (4.3)	30 (1.2)	12 (1.1)	14 (1.6)	4 (1.0)		
Grade						0.030	0.060
I	97 (1.3)	38 (1.6)	18 (1.6)	14 (1.6)	6 (1.5)		
II	1204 (16.4)	342 (14.2)	166 (14.8)	110 (12.6)	66 (16.2)		
III	5981 (81.4)	2013 (83.6)	931 (82.8)	746 (85.2)	336 (82.4)		
IV	69 (0.9)	16 (0.7)	10 (0.9)	6 (0.7)	0 (0.0)		
Regional nodes positive						<0.001	<0.001
0	4047 (55.1)	1670 (69.3)	798 (70.9)	586 (66.9)	286 (70.1)		
1–5	2338 (31.8)	616 (25.6)	278 (24.7)	238 (27.2)	100 (24.5)		
≥6	966 (13.1)	123 (5.1)	49 (4.4)	52 (5.9)	22 (5.4)		
Radiation						<0.001	<0.001
No/unknown	4890 (66.5)	1797 (74.6)	850 (75.6)	648 (74.0)	299 (73.3)		
Yes	2461 (33.5)	612 (25.4)	275 (24.4)	228 (26.0)	109 (26.7)		
Chemotherapy						<0.001	<0.001
No/unknown	1825 (24.8)	325 (13.5)	160 (14.2)	114 (13.0)	51 (12.5)		
Yes	5526 (75.2)	2084 (86.5)	965 (85.8)	762 (87.0)	357 (87.5)		

^a^ P1 value: comparison of the mastectomy only and all reconstruction group.

^b^ P2 value: comparison of the implant, autologous, and combined reconstruction groups.

^c^ Unmarried: single (never married), separated, divorced, widowed, or domestic Partner.

**TABLE 2:** Baseline demographic characteristics of patients with triple‐negative breast cancer after propensity score matching

Variable	Mastectomy only	Reconstruction type					
		All	Implant	Autologous	Combined		
	No. (%)	No. (%)	No. (%)	No. (%)	No. (%)	P1^a^	P2^b^
	2409	2409	1125	876	408		
Age, year						0.059	0.223
<40	464 (19.3)	530 (22.0)	262 (23.3)	179 (20.4)	89 (21.8)		
40–60	1568 (65.1)	1506 (62.5)	689 (61.2)	561 (64.0)	256 (62.7)		
>60	377 (15.6)	373 (15.5)	174 (15.5)	136 (15.5)	63 (15.4)		
Race recode						0.798	0.041
White	1876 (77.9)	1878 (78.0)	887 (78.8)	659 (75.2)	332 (81.4)		
Black	401 (16.6)	390 (16.2)	163 (14.5)	169 (19.3)	58 (14.2)		
Other	132 (5.5)	141 (5.9)	75 (6.7)	48 (5.5)	18 (4.4)		
Marital status						0.692	0.322
Married	1609 (66.8)	1595 (66.2)	743 (66.0)	567 (74.7)	285 (69.9)		
Unmarried^c^	800 (33.2)	814 (33.8)	382 (34.0)	309 (35.3)	123 (30.1)		
Histologic type						0.378	0.750
Ductal	2127 (88.3)	2106 (87.4)	980 (87.1)	766 (87.4)	360 (88.2)		
Other	282 (11.7)	303 (12.6)	145 (12.9)	110 (12.6)	48 (11.8)		
Stage						0.583	0.358
I	750 (31.1)	791 (32.8)	376 (33.4)	282 (32.2)	133 (32.6)		
II	1276 (53.0)	1237 (51.3)	593 (52.7)	433 (49.4)	211 (51.7)		
III	349 (14.5)	351 (15.6)	144 (12.8)	147 (16.8)	60 (14.7)		
IV	34 (1.4)	30 (1.2)	12 (1.1)	14 (1.6)	4 (1.0)		
Grade						0.376	0.284
I	27 (1.1)	38 (1.6)	18 (1.6)	14 (1.6)	6 (1.5)		
II	332 (13.8)	342 (14.2)	166 (14.8)	110 (12.6)	66 (16.2)		
III	2039 (84.6)	2013 (83.6)	931 (82.8)	746 (85.2)	336 (82.4)		
IV	11 (0.5)	16 (0.7)	10 (0.9)	6 (0.7)	0 (0.0)		
Regional nodes positive						0.679	0.438
0	1649 (68.5)	1670 (69.3)	798 (70.9)	586 (66.9)	286 (70.1)		
1–5	642 (26.7)	616 (25.6)	278 (24.7)	238 (27.2)	100 (24.5)		
≥6	118 (4.9)	123 (5.1)	49 (4.4)	52 (5.9)	22 (5.4)		
Radiation						0.766	0.753
No/unknown	1787 (74.2)	1797 (74.6)	850 (75.6)	648 (74.0)	299 (73.3)		
Yes	622 (25.8)	612 (25.4)	275 (24.4)	228 (26.0)	109 (26.7)		
Chemotherapy						0.197	0.415
No/unknown	294 (12.2)	325 (13.5)	160 (14.2)	114 (13.0)	51 (12.5)		
Yes	2115 (87.8)	2084 (86.5)	965 (85.8)	762 (87.0)	357 (87.5)		

^a^ P1 value: comparison of the mastectomy only and all reconstruction group.

^b^ P2 value: comparison of the implant, autologous, and combined reconstruction groups.

^c^ Unmarried: single (never married), separated, divorced, widowed, or domestic Partner.

### 3.2 OS and BCSS outcomes

**FIGURE 2:**
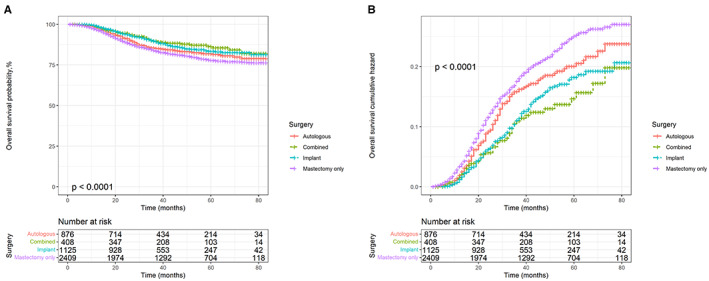
Kaplan–Meier survival and cumulative hazard curves of overall survival according to surgery methods. (A) Overall survival probability. (B) Cumulative hazard curves.

**FIGURE 3:**
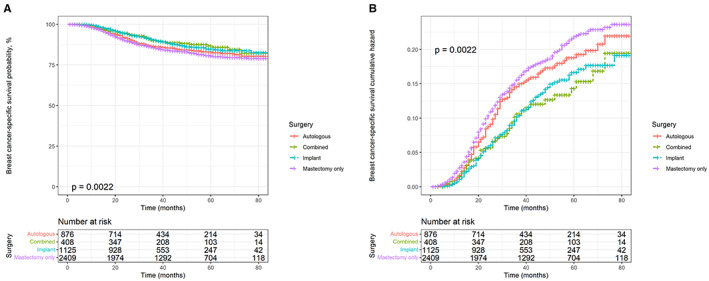
Kaplan–Meier survival and cumulative hazard curves of breast cancer‐specific survival according to surgery methods. (A) Breast cancer‐specific survival probability. (B) Cumulative hazard curves.

Kaplan–Meier and cumulative hazard curves were generated per surgery methods to estimate the overall and cancer‐specific survival of patients with TNBC. As shown in Figure 2, all three reconstruction methods had higher OS and lower risk than mastectomy alone (*p* < 0.0001). Among the three reconstruction methods, autologous reconstruction had the lowest survival rate and the highest risk, while the other two reconstruction methods had little difference in survival benefits. In addition, the Kaplan–Meier and cumulative hazard curves of BCSS shown in Figure 3 were similar to those of OS (*p* = 0.0022).

Univariate Cox analysis showed significant differences between subgroups in terms of surgery, race, marital status, stage, grade, number of positive regional nodes, radiation, and chemotherapy, regardless of OS or BCSS. Considering that the correlation between features may lead to bias, we performed a multivariate Cox analysis. The results revealed that there were five independent risk factors related to OS, while there were only four independent risk factors related to BCSS. Compared with the mastectomy subgroup, all three reconstruction subgroups showed better OS and BCSS, especially implant reconstruction and combined reconstruction. In terms of race, multivariate analysis between blacks and whites showed no statistical difference in the OS (Hazard ratio [HR], 0.919; 95% Confidence Interval [CI], 0.763–1.108; *p* = 0.378) and BCSS (HR, 0.915; 95% CI, 0.752–1.114; *p* = 0.378). Although there was also no significant difference between BCSS (HR, 0.639; 95% CI, 0.405–1.009; *p* = 0.054)of other races and blacks, their OS (HR, 0.593; 95% CI, 0.381–0.925; *p* = 0.021) was better than blacks. Single, divorced, separated, or widowed people demonstrated worse OS (HR, 1.266; 95% CI, 1.088–1.474; *p* = 0.002) and BCSS (HR, 1.205; 95% CI, 1.026–1.415; *p* = 0.023) than those who were married. As expected, the data showed that tumor stage and the number of positive regional lymph nodes were negatively correlated to prognosis (Tables 3 and 4).

**TABLE 3:** Univariate and multivariable Cox analysis of overall survival in patients with triple‐negative breast cancer

Variable	Univariate		Multivariate	
	HR (95% CI)	*p*	HR (95% CI)	*p*
Surgery				
Mastectomy only	Reference		Reference	
Autologous	0.848 (0.696–1.034)	0.104	0.755 (0.618–0.921)	0.006
Implant	0.676 (0.556–0.823)	<0.001	0.666 (0.547–0.810)	<0.001
Combined	0.611 (0.448–0.833)	0.002	0.622 (0.456–0.848)	0.003
Age, year				
<40	Reference		Reference	
40–60	0.949 (0.789–1.142)	0.580	0.980 (0.812–1.182)	0.828
>60	1.233 (1.978–1.554)	0.077	1.261 (0.996–1.598)	0.054
Race recode				
Black	Reference		Reference	
White	0.767 (0.640–0.919)	0.004	0.919 (0.763–1.108)	0.378
Other	0.419 (0.270–0.651)	<0.001	0.593 (0.381–0.925)	0.021
Marital status				
Married	Reference		Reference	
Unmarried	1.521 (1.313–1.762)	<0.001	1.266 (1.088–1.474)	0.002
Histologic type				
Ductal	Reference		Reference	
Other	1.055 (0.843–1.320)	0.640	1.098 (0.875–1.379)	0.419
Stage				
I	Reference		Reference	
II	2.305 (1.821–2.919)	<0.001	1.883 (1.460–2.428)	<0.001
III	9.098 (7.159–11.561)	<0.001	4.418 (3.245–6.014)	<0.001
IV	18.778 (12.906–27.321)	<0.001	10.423 (6.913–15.714)	<0.001
Grade				
I	Reference		Reference	
II	1.328 (0.615–2.869)	0.471	0.777 (0.355–1.701)	0.528
III	1.578 (0.749–3.324)	0.230	0.890 (0.416–1.907)	0.765
IV	2.939 (1.066–8.105)	0.037	2.786 (1.000–7.761)	0.050
Regional nodes positive				
0	Reference		Reference	
1–5	3.101 (2.638–3.646)	<0.001	1.984 (1.643–2.396)	<0.001
≥6	10.409 (8.489–12.763)	<0.001	3.774 (2.893–4.922)	<0.001
Radiation				
No/unknown	Reference		Reference	
Yes	2.276 (1.965–2.636)	<0.001	0.944 (0.794–1.123)	0.516
Chemotherapy				
No/unknown	Reference		Reference	
Yes	1.396 (1.097–1.775)	0.007	0.849 (0.657–1.098)	0.212

**TABLE 4:** Univariate and multivariable Cox analysis of breast cancer–specific survival in patients with triple‐negative breast cancer

Variable	Univariate		Multivariate	
	HR (95% CI)	*p*	HR (95% CI)	*p*
Surgery				
Mastectomy only	Reference		Reference	
Autologous	0.900 (0.732–1.107)	0.317	0.800 (0.650–0.985)	0.035
Implant	0.707 (0.576–0.869)	<0.001	0.701 (0.570–0.861)	<0.001
Combined	0.682 (0.497–0.935)	0.017	0.690 (0.503–0.947)	0.021
Age, year				
<40	Reference		Reference	
40–60	0.948 (0.781–1.150)	0.586	0.987 (0.811–1.200)	0.892
>60	1.085 (0.845–1.393)	0.522	1.132 (0.877–1.460)	0.341
Race recode				
Black	Reference		Reference	
White	0.750 (0.620–0.906)	0.003	0.915 (0.752–1.114)	0.378
Other	0.440 (0.280–0.690)	<0.001	0.639 (0.405–1.009)	0.054
Marital status				
Married	Reference		Reference	
Unmarried	1.461 (1.251–1.706)	<0.001	1.205 (1.026–1.415)	0.023
Histologic type				
Ductal	Reference		Reference	
Other	1.037 (0.817–1.315)	0.766	1.109 (0.871–1.411)	0.401
Stage				
I	Reference		Reference	
II	2.413 (1.864–3.123)	<0.001	1.859 (1.410–2.451)	<0.001
III	10.340 (7.979–13.400)	<0.001	4.630 (3.330–6.437)	<0.001
IV	21.999 (14.894–32.493)	<0.001	11.492 (7.493–17.623)	<0.001
Grade				
I	Reference		Reference	
II	1.259 (0.547–2.897)	0.589	0.669 (0.287–1.561)	0.352
III	1.675 (0.749–3.744)	0.209	0.858 (0.377–1.953)	0.715
IV	3.002 (1.009–8.932)	0.048	2.693 (0.895–9.100)	0.078
Regional nodes positive				
0	Reference		Reference	
1–5	3.326 (2.800–3.951)	<0.001	2.045 (1.674–2.499)	<0.001
≥6	11.573 (9.352–14.321)	<0.001	3.904 (2.963–5.145)	<0.001
Radiation				
No/unknown	Reference		Reference	
Yes	2.485 (2.130–2.900)	<0.001	0.984 (0.821–1.179)	0.862
Chemotherapy				
No/unknown	Reference		Reference	
Yes	1.653 (1.260–2.170)	<0.001	0.963 (0.722–1.284)	0.797

### 3.3 Effects of reconstruction methods stratified by age

To further explore the impact of the three reconstruction methods on prognosis, the results of multivariate analysis were stratified by age and shown in Table 5. In patients <40 years old, no reconstruction method could improve the prognosis of patients. All three types of reconstruction improved OS in patients aged 40–60 years, especially combined reconstruction (Adjusted hazard ratio [AHR], 0.646; 95% CI, 0.439–0.950; *p* = 0.026). Only implant reconstruction improved BCSS (AHR, 0.672; 95% CI, 0.514–0.878; *p* = 0.004) in this age group. Furthermore, in patients > 60 years old, both autologous reconstruction (AHR, 0.570; 95% CI, 0.350–0.929; *p* = 0.024) and implant reconstruction (AHR, 0.538; 95% CI, 0.339–0.853; *p* = 0.008) showed better OS, while none of the three reconstruction modalities improved BCSS.

**TABLE 5:** Adjusted risk ratios in triple‐negative breast cancer patients of different age groups

Age, year	Autologous reconstruction		Implant reconstruction		Combined reconstruction	
	AHR (95% CI)	*p*	AHR (95% CI)	*p*	AHR (95% CI)	*p*
<40						
OS	1.014 (0.661–1.553)	0.951	0.795 (0.515–1.228)	0.301	0.519 (0.259–1.040)	0.065
BCSS	1.054 (0.676–1.645)	0.816	0.901 (0.578–1.403)	0.644	0.516 (0.246–1.080)	0.079
40–60						
OS	0.740 (0.572–0.957)	0.022	0.679 (0.527–0.876)	0.003	0.646 (0.439–0.950)	0.026
BCSS	0.784 (0.601–1.021)	0.071	0.672 (0.514–0.878)	0.004	0.713 (0.484–1.051)	0.088
>60						
OS	0.570 (0.350–0.929)	0.024	0.538 (0.339–0.853)	0.008	0.576 (0.249–1.334)	0.198
BCSS	0.583 (0.338–1.007)	0.053	0.612 (0.371–1.008)	0.054	0.784 (0.334–1.840)	0.576

### 3.4 Competitive risk model

**FIGURE 4:**
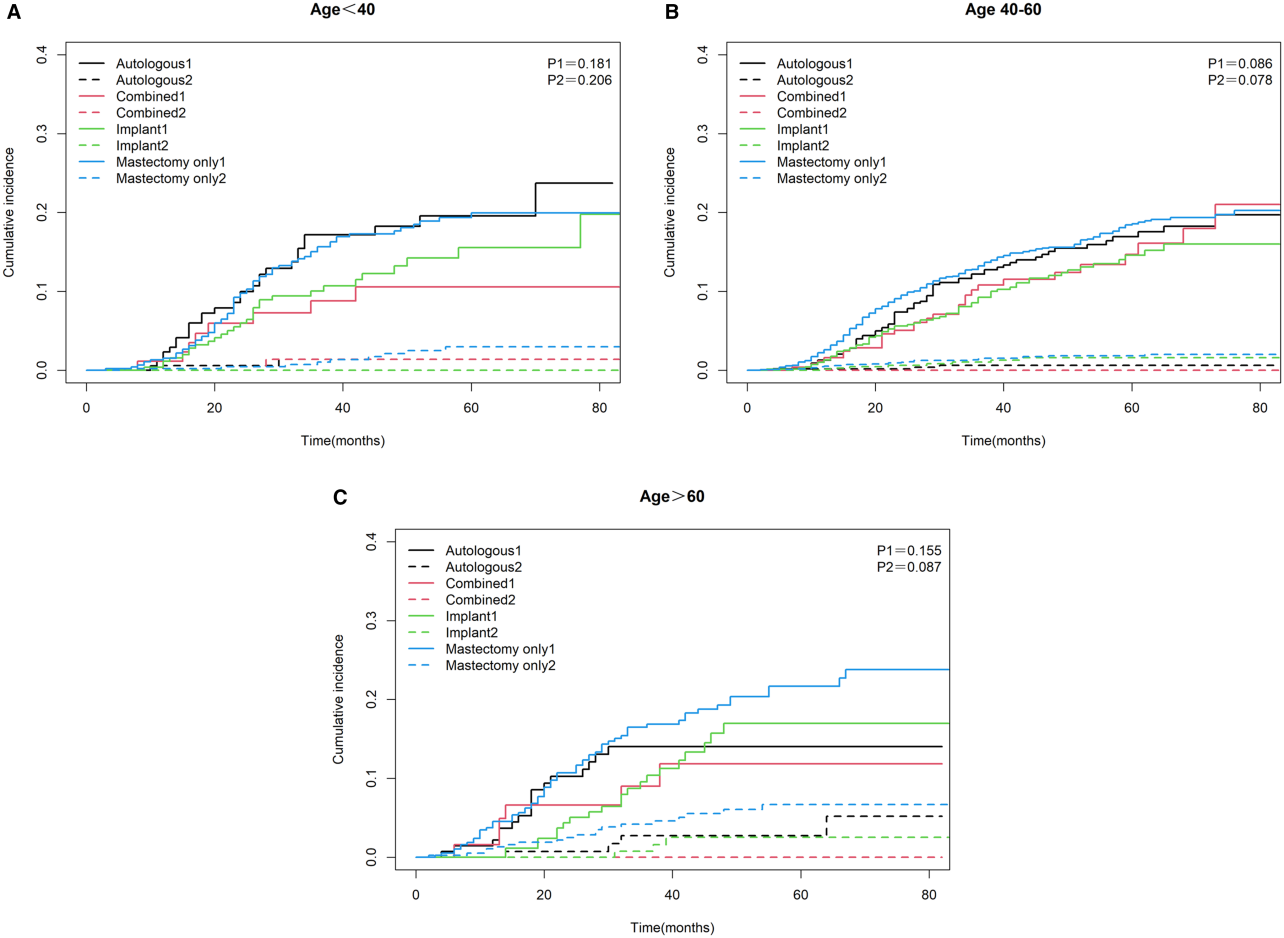
Cumulative mortality curves under competitive risk model. P1: comparison of breast cancer‐specific death between different operations; P2: comparison of non‐breast cancer‐specific death between different operations. (A) Age＜40. (B) Age 40–60. (C) Age＞60. Abbreviations: 1, Breast cancer‐specific death; 2, Non‐breast cancer‐specific death.

We divided the cause of death of TNBC patients into BCSD and NBCSD. The cumulative mortality curves under different surgery methods were drawn as Figure 4. The three graphs showed common characteristics, with a higher cumulative incidence of BCSD than the corresponding NBCSD for each type of surgery in each age group, and BCSD rates were significantly different (*p* = 0.003). Specifically, after controlling the competitive risk factors, the cumulative incidence of BCSD of each reconstruction method was lower than that of mastectomy alone. But the advantages of implant reconstruction and combined reconstruction were the most obvious.

### 3.5 Nomograms

**FIGURE 5:**
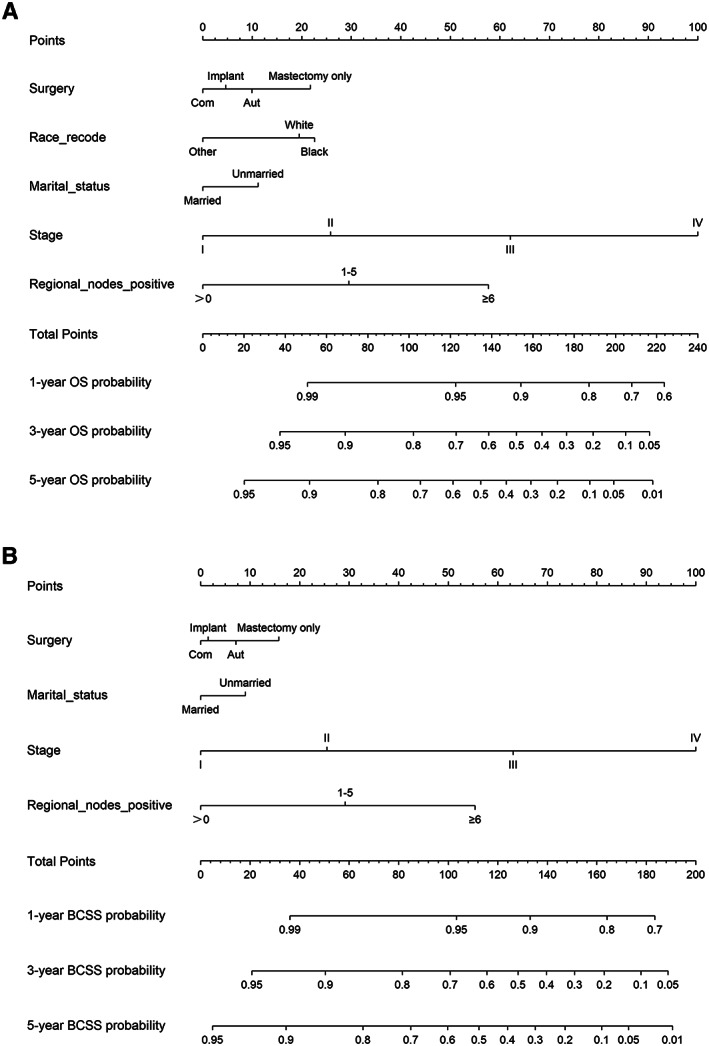
Overall survival (A) and breast cancer‐specific survival (B) nomograms. Abbreviations: Surgery: Com, Combined; Aut, Autologous.

**FIGURE 6:**
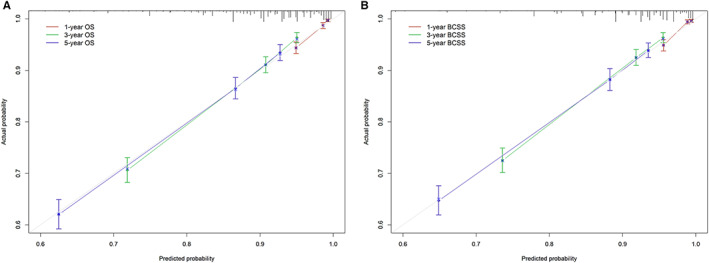
1‐year, 3‐year, and 5‐year nomogram calibration curves. (A) Calibration curves for overall survival. (B) Calibration curves for breast cancer‐specific survival.

The 1‐, 3‐, and 5‐year OS and BCSS probabilities of patients were predicted using nomograms (Figure 5). Based on the above multivariate analysis results, surgery, race, marital status, stage, and the number of positive regional lymph nodes affected the OS of patients. Still, race did not affect the BCSS of patients. Therefore, these metrics were included in the corresponding nomograms. Each item corresponded to a certain score value, and the final total score was used to predict the patients' 1‐, 3‐, and 5‐year prognostic survival probabilities. The C‐indexes of nomograms for OS and BCSS were 0.762 and 0.77, respectively (Figure 6). The calibration curves also showed the accuracy of the survival prediction model.

## 4 DISCUSSION

Through the SEER database, we obtained the data of patients with TNBC, and divided the patients into multiple subgroups according to the different surgeries. This was done to explore the impact of IBR surgery on the survival benefit of patients with TNBC. Kaplan–Meier and cumulative hazard curves suggested that all three reconstruction methods increased the patients' OS and BCSS while reducing their risk. Other survival analyses showed that the survival benefits of reconstructive surgery varied at different ages. Furthermore, implant reconstruction could improve OS and BCSS, but autologous reconstruction and combined reconstruction could only improve OS.

Studies have confirmed that patients who underwent IBR had a significantly lower risk of death than those who had mastectomy alone.^28^, ^33^ Similarly, a study of 52,249 patients by Jayant Agarwal also found that IBR could improve survival benefits.^29^ Gem M Le et al.^28^ found that patients who underwent implant reconstruction had a lower risk of BC mortality than those who had mastectomy alone, after adjusting for age, years of diagnosis, and other factors. Claudia R Albornoz et al.^20^ found that IBR rates in the United States were increasing every year, and implant reconstruction surpassed autologous reconstruction as the leading reconstruction approach in 2002. Researchers further investigated the biological reasons for improved survival with reconstruction, particularly implant reconstruction. American scholars have confirmed the anti‐breast cancer effect of silicone prostheses in animal experiments.^34^ A qualitative review coupled with a meta‐analysis suggested that breast implants may have had a protective effect against BC.^35^ Additionally, studies have found that mature adipocytes may have a potential role in promoting the progression of BC.^36^, ^37^ Therefore, we hypothesized that it was the additional mature adipocytes from the autologous reconstruction of the surgical area that made the reconstruction less effective than implant reconstruction. Interestingly, the long‐term satisfaction of patients with autologous reconstruction was higher than that of patients with implant reconstruction.^38^ The higher quality of life may account for the superior survival of patients with autologous reconstruction over those with mastectomy alone.

Our study narrows the target population to patients with TNBC and reached the same conclusion. This indicates that even though patients with TNBC have a more aggressive cancer and a greater risk of recurrence and metastasis, they can also have breast reconstruction. Despite this, after stratification by age, the three types of reconstructive surgery are limited in improving the OS and BCSS of patients with TNBC, especially among the young (<40) and elderly (>60) patients. This may be because of the higher degree of BC malignancy and likelihood of relapse and metastasis in young women, and poorer physical fitness of elderly women, resulting in limited advantage of reconstruction for both groups. Moreover, the small sample size of the group, <40 years old, may also make the results unreliable. However, people aged 40–60 opted for reconstruction at a higher rate than the other two age groups, with a lot of improvements in OS and BCSS, especially in implant reconstruction. To eliminate the effects of other causes of death, we construct a competitive risk model. Results show that the cumulative incidence of BCSD with any reconstruction method is lower than with mastectomy alone. The two reconstruction methods involving implant reconstruction are relatively superior. This is basically consistent with the above Cox analysis.

Compared with previous studies, the present study has several advantages. First, we used data mining to obtain big data based on the population from SEER. The SEER data are national, effective, and make our research larger and more reliable.^39^ Second, previous studies on reconstruction after mastectomy for BC involves all types of patients with BC. However, our study focuses on patients with TNBC only, which makes up for the lack of research data on patients with TNBC. Third, due to the differences in past medical history‐such as hypertension and diabetes‐in different age groups, patients are stratified according to age in this study, and the influence of confounding factors is adjusted as much as possible. Fourthly, this study adopts the method of multi‐model joint analysis (Cox risk regression model, competitive risk model and survival prediction model) to make the results more comprehensive. Inevitably, however, the study has limitations. Firstly, retrospective studies differ from prospective studies since they have inherent bias. Moreover, the SEER database lacks information on comorbidities such as cardiovascular and pulmonary diseases which can affect the prognosis of patients. This leads to inaccurate results in our study. Although we have stratified patients by age, the specific incidence of the disease differs among age groups. Moreover, our data are from patients in the United States, so the results are only applicable to the US; whether they are applicable to other countries remains to be studied. Lastly, the SEER database only records IBR data but not DBR data, we could not know whether the patient underwent DBR after surgery, affecting our analysis of the prognosis in included patients.

This study provides suggestions for patients with TNBC and doctors on the feasibility and choices for reconstruction after mastectomy. The survival prediction model can also help clinicians quantify the survival benefits of TNBC patients undergoing different surgeries, so as to develop personalized treatment strategies. Reconstruction can indeed improve the OS and BCSS of TNBC patients. Especially, implant reconstruction has the most obvious advantage. In addition, the best reconstruction mode should be selected according to different ages, which requires constantly updated guidelines.

## AUTHOR CONTRIBUTIONS

The authors' responsibilities were as follows: Luyao Dai and Hanxiao Cui designed the study; Xiaobin Ma managed the study; Yuanhang Bao extracted the data; Liqun Hu and Meng Wang performed the analyses; Luyao Dai, Shuai Lin and Zhangjian Zhou interpreted the evidence and wrote the manuscript; Hao Wu, Huafeng Kang and Xiaobin Ma revised the article. All authors agreed to be accountable for the work.

## FUNDING INFORMATION

This study was supported by the National Natural Science Foundation of China (No. 82103129), Basic Research Program of Natural Science Foundation of Shaanxi Province (No. 2021JQ‐422), Prior Science and Technology Program for Overseas Chinese Talents of Shaanxi Province (No. 2020–015), and Key Research and Development Program of Shaanxi Province (No. 2022KW‐01).

## CONFLICT OF INTEREST

The authors declare no conflict of interest.

## Data Availability

Data for this study were collected from the SEER program of the National Cancer Institute. This is a public database with the following links: https://seer.cancer.gov.
